# Women Students at Charing Cross Hospital

**Published:** 1915-07-24

**Authors:** 


### WOMEN STUDENTS AT CHARING CROSS HOSPITAL.

An important decision has been taken by the council of the Charing Cross Hospital Medical School. It has been decided to admit female medical students to the courses of study held at this school. This new departure has been prompted not solely by the falling off in numbers of the present generation of medical students of the male sex, but also by the fact that at the London School of Medicine for Women (Royal Free Hospital) the number of applicants for a medical training is greater than can conveniently be coped with by the authorities at that school. The somewhat glowing accounts recently published of the prospects of women who successfully pursue the full course of study for a medical qualification have evidently borne fruit. Time alone can show how far these encouraging views (or the pessimistic opinions of not a few general practitioners) are justified. Meanwhile the fees of new women students will be a welcome addition to the depleted exchequers of some of the London hospitals, for is it to be supposed that Charing Cross Hospital, although first in the field, will have the monopoly of the overflow from the Royal Free Hospital? However, Charing Cross Hospital is in somewhat of a unique position in the matter, and the suggestion may be made to the authorities to consider the desirability, by a gradual process, of replacing its male medical students by women altogether. There is much to be said for this proposal, to which we hope to return in an early issue.

The place in the general community of a largely augmented body of women medical practitioners is not now a matter for profitable speculation—a great many still more important changes are foreshadowed for the next quinquennium. But the mixing of male and female students of medicine in our metropolitan hospitals is an event which justly deserves more than passing notice.

Twenty-five years ago—we need hardly go further back than that—the combination would have been regarded as quite impracticable; fifteen years ago, even, the proposal would probably have been dismissed as unsuitable for the Metropolis. Now it would be difficult to raise serious objections against the mingling of the sexes in the class rooms and wards of our hospitals. Rowdyism in lectures is a thing of the past, and any exhibition of high spirits at smoking concerts, or at séances of the Dr. Dowie type, will, we believe, be totally unaffected by the new conditions.

The growing phalanx of women medical students in the past have had one attribute in common—they have been "terribly in earnest." The wider opening of the examination portals has allowed to qualify many women who might not have been quite equal to the degrees of the London University. Now that candidates will be so much more numerous, will the earnestness of the average woman student be maintained at so high a level as before? If so, and we see nothing yet to suggest otherwise, the male members of medical classes will doubtless be spurred on to make efforts which shall maintain their alleged superiority. Would a defeated prizeman try to nullify his want of success by proposing marriage to the victorious lady? The humorous aspect of such a proposal must not conceal from us the possibilities of its occurrence, but the effect of such marriages, did they occur, on medical practice is a large and important question towards which attention may have to be directed when the conclusion of peace and the war's permanent effect upon the position of the woman doctor are accomplished facts for all concerned.

